# Identification and Characterization of the Diverse Stress-Responsive *R2R3-*RMYB Transcription Factor from *Hibiscus sabdariffa* L.

**DOI:** 10.1155/2017/2763259

**Published:** 2017-10-18

**Authors:** Bahaeldeen Babikar Mohamed, Beenish Aftab, Muhammad Bilal Sarwar, Bushra Rashid, Zarnab Ahmad, Sameera Hassan, Tayyab Husnain

**Affiliations:** ^1^Center of Excellence in Molecular Biology, University of the Punjab, 87-West Canal Bank Road, Thokar Niaz Baig, Lahore 53700, Pakistan; ^2^National Center for Research (NCR), Ministry of Sciences and Telecommunications, Khartoum, Sudan

## Abstract

Various regulatory proteins play a fundamental role to manage the healthy plant growth under stress conditions. Differential display reverse transcriptase PCR and random amplification of cDNA ends (RACE) was used to explore the osmotic stress-responsive transcripts. We identified and characterized the salt stress-responsive R2R3 type RMYB transcription factor from *Hibiscus sabdariffa* which has an open reading frame of 690 bp, encoding 229 long chain amino acids. In silico analysis confirmed the conserved R2 and R3 domain as well as an NLS-1 localization site. The deduced amino acids of RMYB shared 83, 81, 80, 79, 72, 71, and 66% homology with *Arabidopsis thaliana*, *Glycine max*, *Oryza sativa*, *Zea maize*, *Malus domestica*, *Populus tremula* × *Populus alba*, and *Medicago sativa* specific MYB family, respectively. We observed the gene upregulation in stem, leaf, and root tissue in response to abiotic stress. Furthermore, RMYB gene was cloned into plant expression vector under CaMV35S promoter and transformed to *Gossypium hirsutum*: a local cotton cultivar. Overexpression of RMYB was observed in transgenic plants under abiotic stresses which further suggests its regulatory role in response to stressful conditions. The RMYB transcription factor-overexpressing in transgenic cotton plants may be used as potential agent for the development of stress tolerant crop cultivars.

## 1. Introduction

Drought and salinity are the most devastating environmental stresses, which cause major reductions in crop productivity and quality. Worldwide, about 20% of the total cultivated land and 33% of irrigated agricultural land are affected by salinity with 10% annual increase due to saline water supply and poor agricultural practices [1]. These statistics make salinity a mighty abiotic stress factor that limits the plant growth particularly root to shoot ratio due to limited photosynthetic activity. Understanding the salinization impact on root tissue is critical, as it directly bears the excessive ion exchange imbalance in surrounding areas and it also performs other numerous vital functions like holding of the plant in the soil, water, and nutrient uptake and ionic homeostasis [2]. Regulation of gene expression under salinity stress includes a wide array of mechanisms that are used by plants to up or downregulate the production of specific gene products (mRNA to protein). These genes involve three major categories: (i) those that are involved in signaling cascade and in transcriptional control (transcription factor), such as MyC, MAP, and *SOS* kinases phospholipids and transcription factors such as *HSF and CBF/DREB*, MYB, bZIp, bHLH, WRKY, and NAC transcription factors; (ii) those that function directly in the protection of membranes and proteins, such as heat shock protein (*HSPs*) and chaperones, and late embryogenesis abundant (*LEA*) proteins; and (iii) those that are involved in water and ion transport such as aquaporins and ion transporters [3]. The most important regulators of genes and gene clusters are the transcription factors. Their structure of DNA-binding domain is considered for classification into families and subfamilies. Many families like bZIP, WRKY, AP2, NAC, C_2_H_2_ zinc finger, and MYB TFs have been deeply studied to play a regulatory role in a stressful environment.

MYB protein constitutes one of the largest TF groups that are functionally diverse and found in all eukaryotes. Currently, some of the MYB proteins have been identified in *Arabidopsis*, rice (*Oryza sativa*) [4], maize (*Zea mays*), and cotton (*Gossypium hirsutum*) genome [5]. Most of the plant MYB TFs belong to the R2R3-type and play an active role in (1) primary and secondary metabolites, (2) cell fate and identity, (3) developmental processes, and (4) response to biotic and abiotic stresses, while others like R1/2-MYB proteins are mainly involved in regulation of circadian rhythms. R1R2R3-MYB proteins participate in the formation of B-type cyclin, cell-cycle control, and stress tolerance [4].

Over the past decade, the role of different members of MYB has been deeply studied in response to diverse abiotic stress conditions. For instance, AtMYB2 functions in the ABA-mediated drought stress response in *Arabidopsis thaliana* [6]. AtMYB102 is found to be a key regulatory component in response to wounding, osmotic and salinity stress, and ABA application. In addition, AtMYB96 modulates ABA signaling in response to abiotic stresses in *Arabidopsis thaliana* [7]. Overexpression of *AtMYB44* has been observed to enhance the abiotic stress tolerance by maintaining the stomatal closure mechanism in transgenic *Arabidopsis thaliana* [8].

*Hibiscus sabdariffa* L. (English name: Red sorrel, Roselle; Arabic name: Karkade) belonging to the Malvaceae family is an annual plant with basic chromosomal number *x* = 18 (4*n* = 72). It is mostly grown in tropical and subtropical regions of Africa. Its fleshy calyx (sepals) is a good source of natural antioxidants (anthocyanin and protocatechuic acid) that protects the membrane from damage by free radicals especially lipid peroxidation [9]. DDRT-PCR is a reliable technique which is primarily based on reverse transcriptase polymerase chain reaction [10]. It has been used to isolate differentially expressed genes from different plant species under abiotic stresses [11, 12]. In this study, we have reported a novel nuclear-localized R2R3-type RMYB gene isolated from *Hibiscus sabdariffa* L. under salt stress. Up till now, MYB TFs have been identified and isolated from some land plants, but this study consists of the first report regarding the characterization of MYB TFs from the *Hibiscus sabdariffa* L. roots in response to salt stress. Overexpressing gene in transgenic plants suggests its putative role in the regulation of stress tolerance mechanism.

## 2. Materials and Methods

### 2.1. Plant Material, Stress Treatment, and RNA Isolation

Seeds of *Hibiscus sabdariffa* L. cultivar (Bulk Rahad) were grown in plastic pots (25 × 30 cm) with composite soil mixture (peat, soil, and sand) in equal proportion (1 : 1 : 1) in the greenhouse at 25 ± 2°C with relative humidity near 50% [9]. One month after germination, 100 mM NaCl was applied to the experimental plants. A control group of plants watered with normal tap water was also maintained as controls. After stress application, roots of treated and control plants were sampled and immediately frozen in liquid nitrogen and total RNA isolation was carried out by using phenol-based method [13]. Agarose gel electrophoresis (0.8%) was done to check the integrity of total RNA, and quantification was done by using the Nanodrop, ND-1000 spectrophotometer (NanoDrop Technologies Inc.). *DNase*1 (Sigma number AMPD1-1KT) treatment was done to remove the DNA contamination.

### 2.2. RMYB Gene Identification

Differential display of mRNA was performed as previously described [10], with minor modifications. Total RNA (1 *μ*g) was reverse transcribed with an anchored oligo-dT primer by using RevertAid™ H Minus First-Strand cDNA Synthesis Kit (Fermentas, Burlington, ON) according to the manufacturer's protocol. DDRT-PCR was carried out with 15 arbitrary primers in combination with 11 anchoring primers randomly (Supplementary Table 1 available online at https://doi.org/10.1155/2017/2763259) in 25 *μ*l volume containing 2.5 units (0.5) *μ*l of Taq Polymerase (Invitrogen), 1 *μ*l of each primer (10 picomole), 0.5 mM dNTPs, 2.5 *μ*l 10 × PCR buffer, 500 ng (1 *μ*l) cDNA, and 1 *μ*l (50 mM) MgCl_2_. The cycling conditions were: initial denaturation at 95°C for 2 min followed by 35 cycles of denaturation at 95°C for 45 s, annealing at 42°C for 1.30 min, extension at 72°C for 45 s, and a final elongation step at 72°C for 10 min. The amplified products were run on 16% denatured polyacrylamide gel as mentioned by Maqbool et al. [3]. Reproduced amplicon was cloned into pJET vector and sequenced by using the ABI Prism Dye Terminator kit and ABI model 3100 automated DNA Sequencer (Applied Biosystems, Foster City, CA) while homology study was done with online BLAST tool (http://blast.ncbi.nlm.nih.gov/Blast.cgi). The potential amplicon was further amplified from ends by using the GeneRacer kit (Invitrogen Life Technologies, Carlsbad, CA). Total RNA (3 *μ*g) was used to generate RACE ready first-strand cDNA and forward gene-specific primer (5′- GCAGGATCGAGACCTTGGCGTCTCA-3′), reverse primer (5′- TCAGACATCCGCGGACGGTGCTTTA-3′), and gene racer primer (5′-CGACTGGAGCACGAG-GACACTGA-3′) were used to generate the 5′ end of the gene. The PCR profile was set as initial denaturation and cycle one at 94°C for 2 min, annealing at 67°C for 30 s, and extension at 68°C for 1 min. After every five subsequent cycles, the annealing temperature was decreased by 1°C until 62°C temperature was achieved and additional 30 cycles at an annealing temperature of 62°C were carried out at the end. PCR products were resolved in 1% (*w*/*v*) agarose geland DNA fragment of the expected size was purified, cloned, and sequenced. Amplification of RACE generated full-length gene from genomic DNA was done by using the primers *RMYB-F* (5′TTACTCCCACCTTCTGCAATTT′3) and *RMYB-R* (5′GATTGAGTAGGCACCGAAGTTT′3) then cloned into pJET1.2 vector and sequenced.

Homology studies were carried out by using online BLAST (Basic Local Alignment Search Tool) and Pairwise alignment algorithm (http://www.ebi.ac.uk/emboss/align) programs. Exon/intron boundaries, untranslated regions (UTRs), and poly-A tail regions were located by softberry server (http://www.softberry.com/berry.phtml). The conceptual translation of nucleotide sequence was done by using the Open Reading Frame Finder (ORF) program (http://www.ncbi.nlm.nih.gov/gorf/gorf.html). Molecular weight and subcellular localization were carried out at ExPASy server (http://expasy.org). PSIPRED software was used to predict the RMYB gene secondary structure. Transmembrane helix of the RMYB was calculated by MEMSAT3 and MEMSATVSVM software while the PredictProtein server was used to understand the solvent accessibility of the protein. Multiple sequence alignment was performed by using the CLUSTALW with default parameters through EMBnet (http://www.ch.embnet.org/software/ClustalW.html). Phylogenetic relationship was constructed by using the CLC Genomics Workbench software with R2R3-MYB publicly available amino acid sequence of MYB-like 102 *Arabidopsis thaliana* (NP_567626), transcription factor *MYB*39 of *Oryza sativa* Japonica group (XP_015645522)*, Glycine max MYB* transcription factor *MYB*77 (ABH02890)*, Medicago sativa MYB* transcription factor (XP_003608268)*, MYB* DNA-binding domain superfamily protein from *Zea mays* (DAA63102)*, Malus domestica* (NP_001281023), and *MYB* transcription factor from *Populus tremula* × *Populus alba* (AAN05422). Structural modeling was carried out by homology modeling servers; Phyre2 (http://www.sbg.bio.ic.ac.uk/phyre2/html/page.cgi?id=index), Jigsaw (http://bmm.cancerresearchuk.org/~3djigsaw/), SWISS-MODEL (http://swissmodel.expasy.org/interactive), I-Tasser (http://zhanglab.ccmb.med.umich.edu/I-TASSER/), and RaptorX (http://raptorx.uchicago.edu/). Those models were further inspected by the Phi/Psi Ramachandran plot and 3D ID score was calculated at PROCHECK server (http://services.mbi.ucla.edu/SAVES/). Active binding amino acid residues within SANT domains were predicted by the online metaDBSite database [14]. PyMOL-Educational software (http://www.pymol.org/) was used to visualize the predicted parameters in 3D structure. Motif Scan (http://myhits.isb-sib.ch/cgi-bin/motif_scan) web server was used to study the primary MYB protein for functionally active sites such as N-glycosylation sites, protein Kinases sites, and sites for myristoylation and phosphorylation.

### 2.3. Expression Analysis of RMYB Transcript by Real-Time PCR

The response of RMYB transcript at tissue-specific level (root, leaf, and stem) was studied under drought, salt, and cold stress in the greenhouse. Gene-specific primers RMYB*-*F (5-CACATTCCAAGGCTGGATCT-3), RMYB-R (5-CAACCGGACCATCATCTTCT-3) were used for this purpose. Salt stress was applied as described in the previous section; cold stress was applied by keeping the plants at 4°C, while drought stress was applied by cessation of water while maintaining control plants without any stress treatment to normalize the relative fold data. Total RNA was extracted from leaf, stem, and root tissue of both control and stressed plants as described before. IQ5 cycler (Bio-Rad, Hercules, CA) was used with IQ™ SYBR_Green Super-mix dye (Bio-Rad), while qPCR cycling conditions were set an initial denaturation at 95°C for 5 min followed by 40 cycles of denaturation at 94°C for 30 s, annealing at 60°C for 30 s with extension at 72°C for 40 s and a final elongation at 72°C for 10 min. Melting curve analysis was performed by continuous monitoring of the fluorescence between 60°C and 95°C with 0.5°C increment after every 30 s. Statistical analysis was performed by using the Relative Expression Search Tool (REST) (http://rest-2009.gene-quantification.info/). *β-Actin* gene expression was used for data normalization. Analysis of variance (ANOVA) was performed by using STATISTICA 8.1 data analysis software to observe the significant difference of RMYB expression in different plant tissues.

### 2.4. Plant Expression Vector Construction and Genetic Transformation

Full-length sequence primers with *Xho*I restriction sites (*Xho*I-F 5′-GGCTCGAGATGTCGAAACCTCCTCCTCCT-3′, *Xho*I-R 5′-GGCTCGAGTTACCTGGGACCGGAAAGC-3′) at 5′ ends were used to amplify the open reading frame of RMYB transcript which introduced the *Xho*I restriction site within up and downstream of the start and stop codon. The amplified product and pCAMBIA-1301 vector were double digested with *Xho*I individually for the development of overhang region and further ligated by using T4 DNA Ligase (Invitrogen) according to the manufacturer's protocol. Cloning and orientation of RMYB transcript were confirmed via restriction digestion and orientation primers. Mature embryos of the local cotton variety CIM-496 were used for the transformation of RMYB gene through *Agrobacterium*-mediated transformation method [15] with some modifications. Nontransformed plants were maintained as control. GUS-positive plants were acclimatized and shifted to a greenhouse for further analysis.

### 2.5. Stress Tolerance and Expression Analysis of Transgenic Plants

Transgenic plants were screened out through PCR by using a forward primer for vector and reverse gene-specific primer. Drought, salt, and cold stress were applied to the transgenic as well as nontransgenic plants after 3 weeks of shifting of plants to the greenhouse. 100 mM NaCl stress was applied for 5 days, while plants were dehydrated up to 5 days to apply the drought stress. For cold stress, the transgenic plants were kept in the cold room at 4°C for 5 h (each stress was applied to a separate group of plants). *β-Actin* gene-specific primers, *β-actin-F* (5′ TGGGGCTACTCTCAAAGGGTTG′3) and *β-actin -R* (5′ TGAGAAATTGCTGAAGCCGAAA ′3), were used as internal control to normalize the data. Gene expression was calculated as a fold-increase relative to the same transcript under control conditions with the comparative Ct method by using the Relative Expression Search Tool (REST) based on the 2^−Δct^. Standard deviation was calculated to show the variation in the replicates.

(1) $$\begin{array}{c} Relative\;expression\;ratio=\frac{{\left(E_{target}\right)}^{Cttarget\;\left(control-sample\right)}}{{\left(E_{ref}\right)}^{Ctref\;\left(control-sample\right)}}. \end{array}$$

## 3. Results

### 3.1. Identification of RMYB Transcript

**Figure 1:**
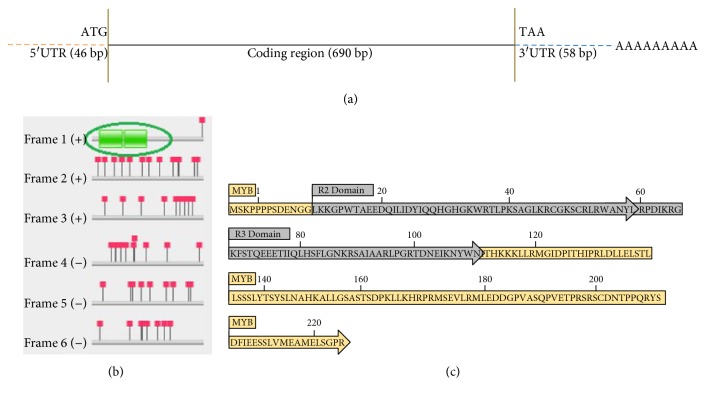
(a) Schematic representation of the full-length RMYB gene layout after RACE analysis. Solid black lines indicate coding region (690 bp), orange color dotted lines indicate 5′ UTR region, and blue dotted lines indicate 3′ UTR region while the sequence of AAAAAA indicates the poly-A tail region of the translated product of the gene. (b) The green box represents the two active SANT/MYB domains in the cDNA sequence as predicted by the Pfam protein family database. (c) The complete schematic presentation of the translated product of RMYB gene. The gray colored amino acids represent the R2R3 MYB DNA-binding domains.

Different combinations of anchored and arbitrary primers generated a number of transcripts after DDRT-PCR. Out of those transcripts, only 5 potential amplicons ranging from 200 to 600 bp showed differential existence consistently. Therefore, those transcripts were isolated, cloned, sequenced, analyzed and, submitted as potential salt-responsive ESTs in NCBI GenBank database with accession numbers [JK757799.1, JK757800.1, JK757801.1, JK757802.1, and JZ152799.1]. BLAST showed significant homology with different stress-responsive genes in multiple plant species such as *Vitis vinifera* cultivar Danuta VINE-1 repeats element, gag-pol polyprotein gene, *Glycine max* F-box protein SKIP14-like, *Gossypium hirsutum* mRNA of *MYB* protein, putative retrotransposon copia, and transposon *GORGE3-like*. Transcript accession number [GenBank: JZ152799] did not show significant homology with any of the functional proteins. The transcript number JK757801.1 encoding myeloblastosis protein was amplified from 5′ and 3′ by RACE, amplifying the extra 435 bp nucleotides by adding up to the previous 225 bp long EST. The newly generated full-length amplicon was named as RMYB which encodes 794 bp long amplicon from cDNA template (GenBank: KC145282), while 690 bp long amplicon was obtained from genomic DNA source (Figure 1(a)).

### 3.2. Sequence Analysis

**Figure 2:**
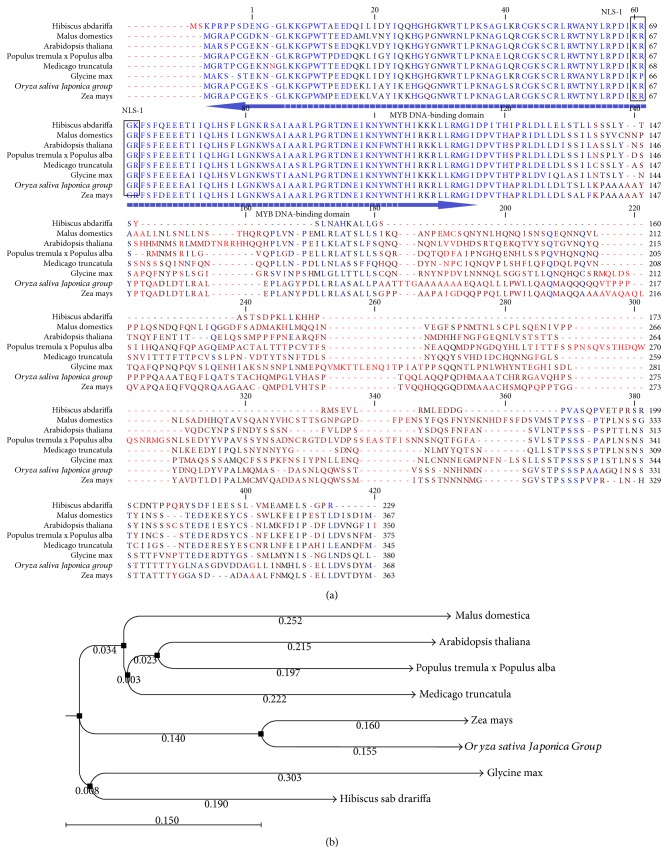
(a) Multiple sequence alignment of RMYB gene protein with other MYB gene sequences. Conserved residues are in blue color. Active functional domain span is represented by an arrow. The box covered portion represents the NLS-1. (b) Distance-based and neighbor-joining tree correlated complete RMYB amino acid sequence to full-length MYB amino acid sequences from different plant species. Bootstrap number on each node represents the significance level while the arrow symbol points out the studied species.

Comparative analysis of the genomic DNA and cDNA sequences of RMYB showed that the transcribed product was intron free. We found 46 bp in the 3′ UTR region and 58 bp in 5′ UTR region downstream the termination codon (TAA). Total 9 bp encompassed the poly-A tail region spanning from 785 to 794 bp on the 3′ end of the cDNA template. Analysis of active MYB/SANT domains in the cDNA sequence through Pfam protein database is shown in Figure 1(b). Translated protein of RMYB gene is composed of 229 amino acids with 5.13 pI and 56.9 kDa. More than 75% sequence similarity was observed with different plant species including gymnosperms and angiosperms with active *MYB* DNA-binding domains (Figure 1(c)). PSIPRED secondary structure prediction showed the presence of 41% helix and 59% loop in the protein sequence of the RMYB gene (Supplementary file 1-S1). PredictProtein server predicted the 72.49% as the exposed area and accessible to the solvents, 7.2% intermediately reactive, while the remaining 20.09% as nonreactive and was buried and inaccessible to the solvents (Supplementary file 1-S2). The regions between 44 to 59 amino acids spanning ATP/GTP-binding site motif at the 47–54aa position as well as C-terminal extracellular region and 61 to 82 amino acids had a C-terminal extracellular region and cytoplasmic N-terminal region, respectively (Supplementary file 1-S3). Moreover, four CK2_PHOSPHO_SITE caseine kinase II at 20–23, 73–76 position, 102–105, and 162–165aa was observed through PROSITE motif analysis. There were three motifs specific for N-myristoylation site at 47–52, 88–93, and 159–164aa position, two sites for PHOSPHO_SITE protein kinase C phosphorylation at 54–56, 195–197aa position, and one for tyrosine kinase phosphorylation at 101 to 109 aa position. Multiple sequence alignment carried out by CLUSTALW algorithm showed 66–83% similarities for amino acid residues with other plant species such as *Arabidopsis thaliana* (83%), *Glycine max* (81%), *Oryza sativa* (*Japonica* group) (80%), *Zea maize* (79%), *Malus domestica* (72%), *Populus tremula* × *Populus alba* (71%), and *Medicago sativa* (66%) (Figure 2(a)). The phylogenetic study revealed closely related *MYB* genes in *Glycine max* and *Zea mays*, while distantly related to *Medicago truncatula* and *Malus domestica.* These characteristics of RMYB specified its relationship with R2R3-*MYB* gene group family (Figure 2(b)).

### 3.3. Structure Prediction and Active Binding Residue Identification

**Figure 3:**
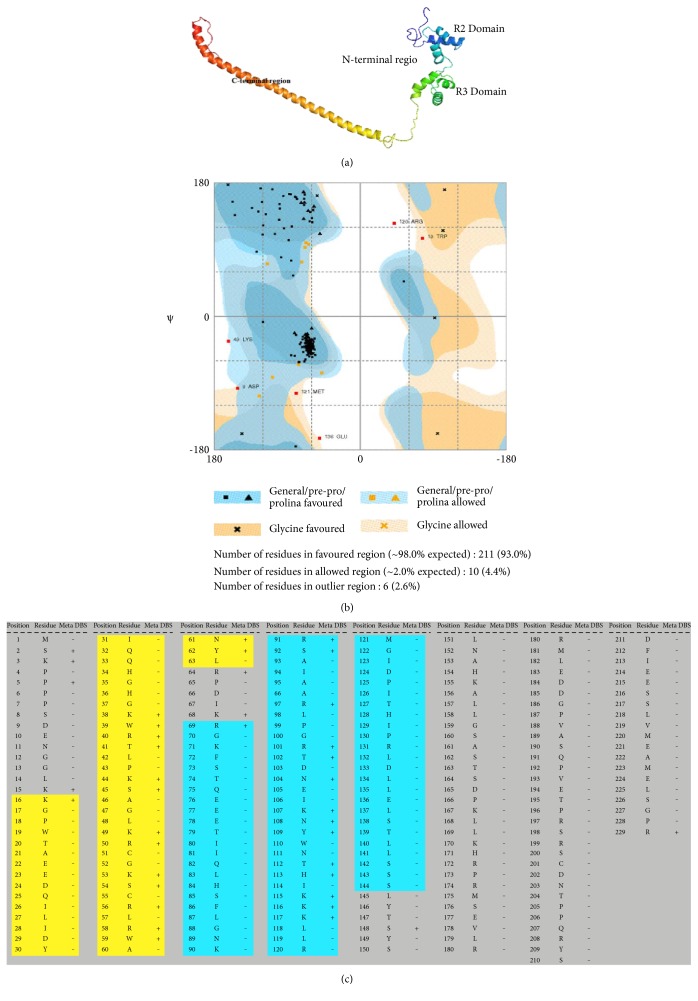
(a) 3D predicted structure for RMYB gene by RaptorX server, with the variable C-terminal region, conserved N-terminal region, and conserved R2 and R3 domains. (b) Ramachandran plot of RMYB gene protein revealing stability parameters. (c) metaDBSite predicted protein binding residue with the nucleic acid. Binding residues are labeled with “+” and nonbinding residues are labeled with “−” and highlighted portion represents the R2R3 domain of the RMYB gene.

**Figure 4:**
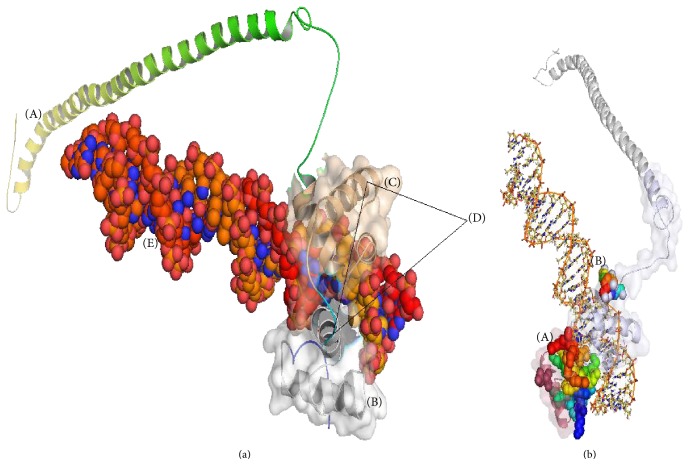
(a) 3D representation of the predicted binding residues of the conserved domain with the DNA (A) variable region of RMYB, (B) R2 domain of RMYB, (C) R3 domain of RMYB, and (D) conserved region among the MYB gene families. (b) Amino acid residues directly involved in binding with DNA in R2 domain (A) predicted amino acid residues for binding with DNA in R3 domain (B).

The 3D structure models of RMYB gene were generated by using different online servers are shown in Supplementary file 1 (S3). Standalone generation of protein structure based on homology modeling was left out as we observed only 50% homology with *Mus musculus* (house mouse) chain A. Crystallized structure of C-*MYB* R2 in the protein data bank (PDB) provided the recommended score to start standalone modeling. The structure was generated by *RaptorX* server and evaluated by using various structural verification software suitable for further studies based on Ramachandran plot and verification of 3D program (Figure 3(a)). The educational version of Pymol visualized the 3D generated structure and PROCHECK analysis further confirmed the stability of the structure (Supplementary file 3). Ramachandran plot analysis revealed more than 93% residues in the favored region, 44% in the allowed region, while only 2.6% in the outlier region of the plot (Figure 3(b)). None of the amino acid residues have Phi/Psi angles in the disallowed region which indicates the high stability of the generated protein structure. Verified 3D verification program also showed the reliable ID score for *RaptorX* generated model. MetaDBSite server analyzed the binding affinity of amino acid residues in the DNA-binding domain. Total 39 residues were predicted to have high binding affinity for the nucleic acid (Figure 3(c)). Among those, 74.3% of the residues was in R2R3 *MYB* binding domain, while the remaining were in the variable C-terminal region (Figures 4(a) and 4(b)).

### 3.4. Expression of RMYB Gene in *Hibiscus sabdariffa* L. in Response to Abiotic Stresses

**Figure 5:**
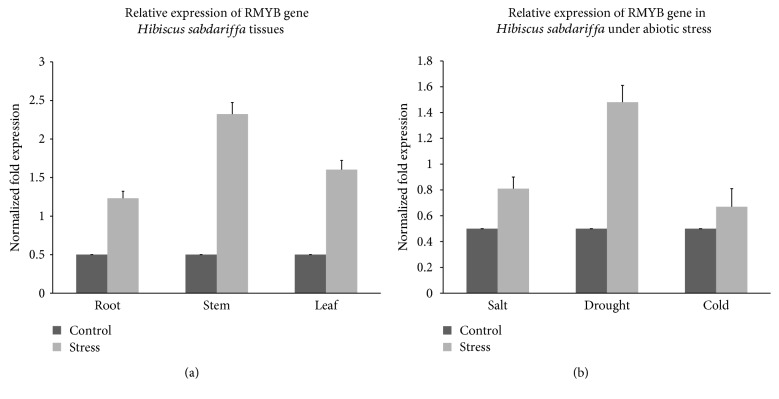
RT-PCR expression analysis of the RMYB gene in *Hibiscus sabdariffa* L. plant tissues under different abiotic stresses. (a) Effect of salt stress on the root, stem, and leaf. (b) Effect of salt, drought, and cold stress on leaves.

An RMYB fragment of 120 bp was amplified by real-time PCR from cDNA of *Hibiscus sabdariffa* L. Gene expression was 2.0, 1.5, and 1.0-fold in the stem, leaf, and root, respectively, under salt stress (Figure 5(a)), while the expression was 8.0, 5.0, and 3.0 times higher in response to drought, salt, and cold stressed roots respectively (Figure 5(b)). Gene expression was compared with control or nonstressed plants and data normalization was carried out by using *β-actin* gene as a housekeeping control.

### 3.5. Gene Cloning and Genetic Transformation

**Figure 6:**
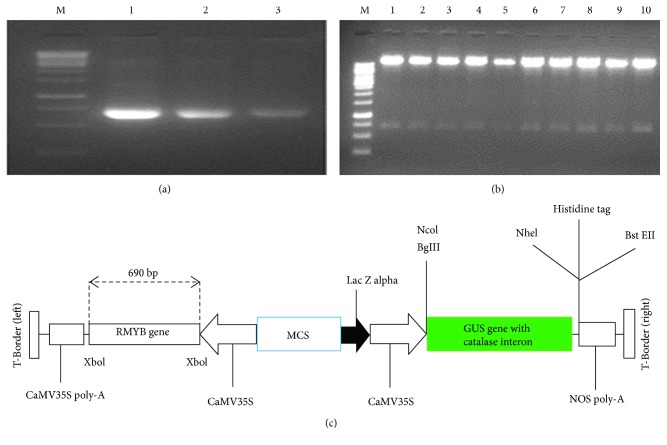
(a) Amplification of the RMYB full-length gene from salt-stressed root of *Hibiscus sabdariffa* L. with *Xho*I restriction sites. Lane M-1 kb marker, lanes 1–3 amplified RMYB product. (b) Cloning confirmation by restriction digestion (c) Schematic representation of RMYB gene in pCAMBIA expression vector in CaMV 35S promoter driven by GUS.

**Figure 7:**
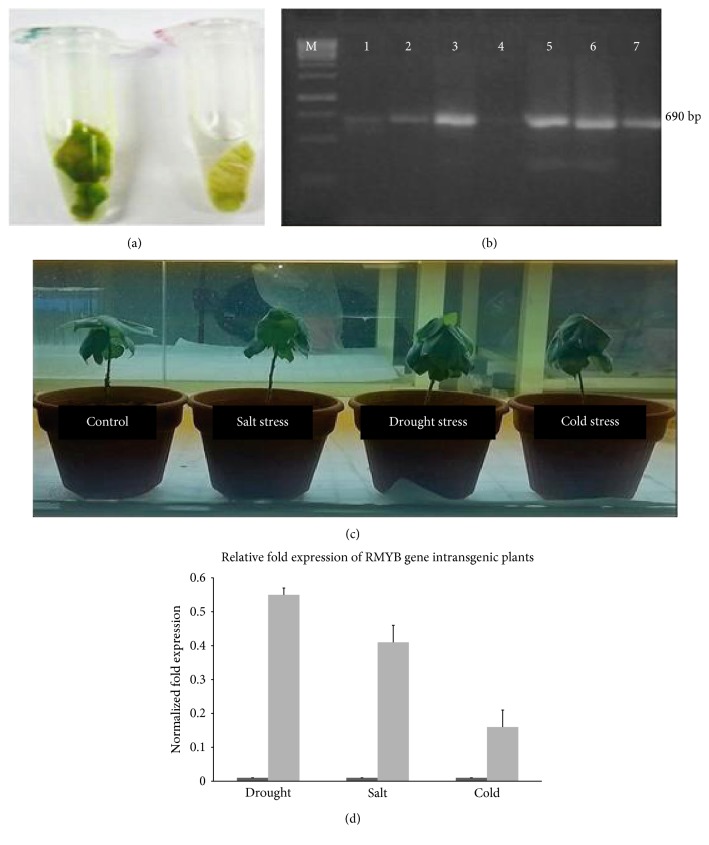
(a) Immunohistochemically GUS assay of the transgenic cotton leaf. (b) PCR amplification of a 690 bp fragment of RMYB gene from transgenic plants, M:1 kb DNA Ladder, lane 1–7 transgenic plants. (c) Transgenic plants in pots under drought, cold, and salt stress. (d) Expression analysis of RMYB gene in transgenic cotton plants under drought, salt, and cold stress. *β-Actin* gene was used as internal control to normalize data and gene expression is indicated as a fold-increase relative to the control condition.

RMYB gene was cloned in the plant expression vector pCAMBIA 1301 (11.837 kb), and full-length primer amplified the complete coding sequence of 690 bp with an insertion of the *Xho*I restriction site sequence before and after the start and stop codon (Figure 6(a)). The ligation was confirmed by restriction digestion (Figure 6(b)). The orientation of insert in the vector was confirmed by orientation primers which yielded the known size of the fragment that was further confirmed by sequencing data, showing 100% homology with the RMYB gene sequence. Complete schematic representation of the cassette of the vector with fragment is shown in Figure 6(c). Complete transformation procedure can be accessed from Supplementary file 1 (S4). Histochemical GUS assay detected the transit GUS gene expression as a blue color in transgenic leaf tissues, while there was no color in the nontransgenic plants' leaves (Figure 7(a)). PCR analysis showed that the genomic DNA of transgenic plants amplified the gene of interest (Figure 7(b)). In this experiment, the overall transformation efficiency was about 0.7%.  Figure 7(c) shows the transgenic plants shifted to the soil and the effect of different abiotic stresses on these plants.

### 3.6. Overexpression of RMYB Gene in Transgenic Plants

Variable response of gene expression was observed in transgenic cotton plants under different abiotic stresses. About 55-fold expression of the RMYB gene was detected under osmotic stress and 41-fold was overexpressed under salt stress. Gene of interest was 15-fold more expressed under the cold stress (Figure 7(d)). This overexpression in transgenic plants was compared to the nontransgenic plants.

## 4. Discussion

TFs play an important role in the regulation of a plant's genetic response, controlling the cascade of genes involved in abiotic stresses. Various studies have been carried out for their identification and characterization. MYB family of transcription factors is a large set of proteins that is mostly involved in an array of functions regarding growth and development, primary and secondary metabolism, cell fate regulation, and response to biotic and abiotic stresses [16]. Differential display profiling is an efficient technique to explore differentially expressed genes under altered growth conditions. The sensitivity of this technique results in good detection of genes present in low abundance (induced or repressed), and only a small amount of starting material is required [10, 17]. Several biotic and abiotic stress-responsive transcripts have been identified from different plant species by this methodology such as tomato, rice, barley, *Arabidopsis*, sunflower, and cotton. However, such type of investigation has yet not been reported in *Hibiscus sabdariffa* L. The RMYB transcript that we have identified and isolated showed more than 70% homology with MYB TF's gene family. Full-length RMYB sequence encoding 229 amino acid long chain with the single open reading frame was retrieved by RACE. In silico analysis revealed the presence of the conserved MYB DNA-binding domains spanning from 16 to 114 amino acid residues at the N-terminus. Recurrence of conserved sequence in the domain is a characteristic feature of MYB TFs (1R, R2R3, 3R, and 4R) on which its nomenclature is based. The RMYB gene revealed its homology to the R2R3 type *MYB* class which is the largest group of plant TFs [18]. R2R3 is thought to be the descendants of R1R2R3-MYB gene ancestor formed by the loss of R1 repeat and subsequent expansion of the gene family. MYB domain is thought to be highly conserved while other regions of R2R3-MYB proteins are mostly variable [6, 19]. The nonconserved diverse sequence at the C-terminal region makes these factors gain wider and distinct features, such as control of development, secondary metabolism, hormonal regulation, and response to biotic and abiotic stresses including drought, salt, wounding, cold, and freezing. We also observed HTH similar repeats in the other MYBs [20]. RMYB gene possesses the NLS-1 signal within the DNA binding domain for transport of proteins into the nucleus like other MYB proteins such as Md*MYB*, Fv*MYB*44 and *GmMYB44* (NLS1-KGRK), *AtMYB59*, and *AtMYB48* (NLS2-RKKAQEKKR) [21–23]. The deduced translated product showed a significant homology with the other crop plants like *Arabidopsis thaliana*, *Zea mays*, *Oryza sativa*, *Glycine max*, *Medicago sativa*. [4]. The coded transcript within the DNA-binding domain of RMYB possesses a highly conserved and evenly distributed residue “w”, which is important for making hydrophobic core and structural stability of the MYB repeats [24, 25].

Phylogenetic analysis of the MYB proteins has been extensively carried out in *Arabidopsis thaliana*, *Oryza sativa*, *Zea mays* and *Populus*, and their evolutionary relationship has been studied systematically [24]. The phylogenetic relationship has grouped the *Hibiscus sabdariffa* L. with *Zea mays* and *Glycine max* specific MYB's families and showed divergence from *Arabidopsis thaliana*, because, a small subset of R2R3 MYB proteins, regulating salt and dehydration genes (i.e., *AtMYB2*) does not contain the first cys residue which makes it impossible to form an S-S bond. Therefore, an alternative mechanism involving nitrosylation of cysteine-S might control its DNA binding activity. These transcription factors also undergo ubiquitination at lysine residues near or at the transcription activation domain, thereby, creating a positive influence on the transcriptional activity. Phosphorylation often has an enhancement effect on the transcriptional activity of MYBs, as observed in *Arabidopsis* and *Nicotiana tabacum* [25, 26]. This divergent relationship reveals that the MYB family underwent a rapid expansion due to gene duplication, sequence deviation, and gene conversion throughout the evolution. Such divergent clades might have been lost with the evolution of *Arabidopsis* lineage or gained along with the development of lineages of rosid and asterid. The subgroups which are closer show that the genes have originated from the same duplication event.

Functional conservation is achieved through the clustering of genes and conserved motifs outside the DNA-binding domain aiding in the identification of different functionally conserved motifs. Redundancy observed at amino acid sequence level among the MYB genes, suggests that, differential/spatial expression of genes causes them to have similar molecular functions, yet, they display variable biological characteristics when mutated. So, developmental biology does not cause the redundancy, instead, during evolution, the genes with closely related functions exist and are maintained together. A single gene is regulated by multiple factors from the same gene family transmitting signals through the same element [19, 26, 27]. Epigenetic modifications and protein-protein interactions can measurably affect the regulatory potential of MYB transcription factors [4]. There is a monomeric DNA binding potential seen *in vitro* in the MYB proteins having two or more MYB repeats. They act as covalently attached dimers, while they make contact with DNA. Dimerization is responsible for the affinity and specificity with which these proteins bind DNA. This outcome diversified the overall functional characteristics, as well as sequence homology, environmental response, and cellular localization [28].

Modeling and interaction studies like metaDBSite analysis further authenticate the functional interaction of RMYB domain with the nucleic acid. About >76% of predicted DNA-protein active binding residues was found within the R2R3 DNA-binding domain. Aspartic acid, arginine, lysine, tryptophan, and serine covered the major portion of active residues within the domain and are potentially considered to be involved in the activities of MYB gene [29]. The relative binding affinities of CASTing, targets to the R2R3-*MYB* domain with kD values and error rates like ACCTAC, ACCAAT, ACCAAA, ACCATA. ACCAAC, ACCACA, and ACCACC, have been confirmed, and the preferred binding sequence was found to be ACCTAC [30]. The role of MYB transcription factor in various physiochemical processes, metabolic regulation, control of cell cycle, and involvement in various defense responses against biotic and abiotic environmental factors has been reported [31]. Variable but significant induction of expression was detected in the stem, root, and leaf tissues under salt stress which shows its critical role in the nucleus. The induction of *TaMYB1* by light under hypoxia, salt stress, and similarly by ABA and PEG application was observed in wheat [32]. Identification and characterization of full-length cDNA sequence of wheat MYB gene *TaPIMP1* against pathogen infection by using RT-PCR, RACE, and its overexpression in transgenic tobacco under drought and salt stress have also been studied [33]. Overexpression of *GhMYB7* in transgenic *Arabidopsis thaliana* enhanced the developing fibers, regulated the secondary cell wall biosynthesis, and acted as the potential transcriptional activator [34]. We also observed the response of RMYB gene toward multiple abiotic stresses and expression was induced in response to drought, salt, and low temperature. It indicates that the RMYB is a TF involved in regulating the response of roselle to low-temperature stress and desiccation regardless of salt stress in the arid and semiarid regions helping to relieve the plants' physiology while bringing it to the normal state. For further elucidation about the activity of MYB gene, overexpression of the RMYB transcript was observed in transgenic cotton plants in response to abiotic stresses. This study would help to cope with the changing external environmental conditions. Our results showed that the gene expression is consistent with the previously reported findings [8, 35]. Characterization of the R2R3 *SbMYB44* transcription factors from the haplotype *Salicornia brachiata* Roxb in response to salt stress has also been carried out which has shown its involvement in multiple abiotic stresses [24]. The stable expression of the drought-specific marker genes like *OsLEA3*, *OsRab16A*, and *OsDREB2A*, in *OsMYB2* overexpressing transgenic plants [36].

In conclusion, the RMYB transcript isolated from *Hibiscus sabdariffa* L. under salt stress is an R2R3 type MYB TF which is involved in stress tolerance mechanism. Overexpression of the RMYB gene in transgenic *Gossypium hirsutum* plants elucidates its potential role to cope with stressful environmental conditions. These transgenic plants could be a potential candidate for breeding and development of tolerant/resistant cultivars.

## Supplementary Material

Supplementary file 1 (A) Secondary structure prediction, loop to helix ratio, solvent accessibility, and transmembrane helix formation prediction. Supplementary file 1 (B) 3D-structural models of RMYB gene by using different software. Supplementary file 1 (C) Verification of 3D model of RMYB gene. Supplementary file 4 (D) Transformation of Gossypium hirsutum L. CV CIM 496. Figure S1: A: Secondary structure prediction of RMYB protein using the PSIPRED software, B: secondary structure of SbMYB44 showing 41% alpha helices and 59% loop, C: solvent accessibility of protein is determined using Predict protein server. Figure S2 A: Schematic diagram of the MEMSAT3 and MEMSATSVM prediction of RMYB showing presence of transmembrane helix (marked by grey region). Supplementary Table 1: List of Arbitrary and Anchored primer used for differentially expressed transcript identification.

## Abbreviations

DDRT-PCR: Differential display reverse transcriptase polymerase chain reaction
RACE: Random amplification of cDNA ends
CaMV: Cauliflower mosaic virus
MYB: Myeloblastosis
TFs: Transcription factors
BLAST: Basic Local Alignment Search Tool
UTRs: Untranslated regions
Poly-A: Polyadenylated A
ANOVA: Analysis of variance
EST's: Expression sequence tags.
