# Supplementary material for: Identification and Characterization of the Diverse Stress-Responsive R2R3-RMYB Transcription Factor from Hibiscus sabdariffa L
Source: Int J Genomics. 2017 Oct 18;2017:2763259. doi: 10.1155/2017/2763259 (PMC5664376; doi:10.1155/2017/2763259)
Supplement: Supplementary file 1 — (A) Secondary structure prediction, loop to helix ratio, solvent accessibility, and transmembrane helix formation prediction. Supplementary file 1 (B) 3D-structural models of RMYB gene by using different software. Supplementary file 1 (C) Verification of 3D model of RMYB gene. Supplementary file 4 (D) Transformation of Gossypium hirsutum L. CV CIM 496. Figure S1: A: Secondary structure prediction of RMYB protein using the PSIPRED software, B: secondary structure of SbMYB44 showing 41% alpha helices and 59% loop, C: solvent accessibility of protein is determined using Predict protein server. Figure S2 A: Schematic diagram of the MEMSAT3 and MEMSATSVM prediction of RMYB showing presence of transmembrane helix (marked by grey region). Supplementary Table 1: List of Arbitrary and Anchored primer used for differentially expressed transcript identification. [file 2763259.f1.docx]

**Supplementary file 1 (A)** Secondary structure prediction, loop to helix ratio, solvent accessibility, and transmembrane helix formation prediction

Figure S1: A: Secondary structure prediction of RMYB protein using the PSIPRED software, B: secondary structure of SbMYB44 showing 41% alpha helices and 59% loop, C: solvent accessibility of protein is determined using Predict protein server.

Figure S2 A: Schematic diagram of the MEMSAT3 and MEMSATSVM prediction of RMYB showing presence of transmembrane helix (marked by grey region),

**S1**

Secondary structure

A

**
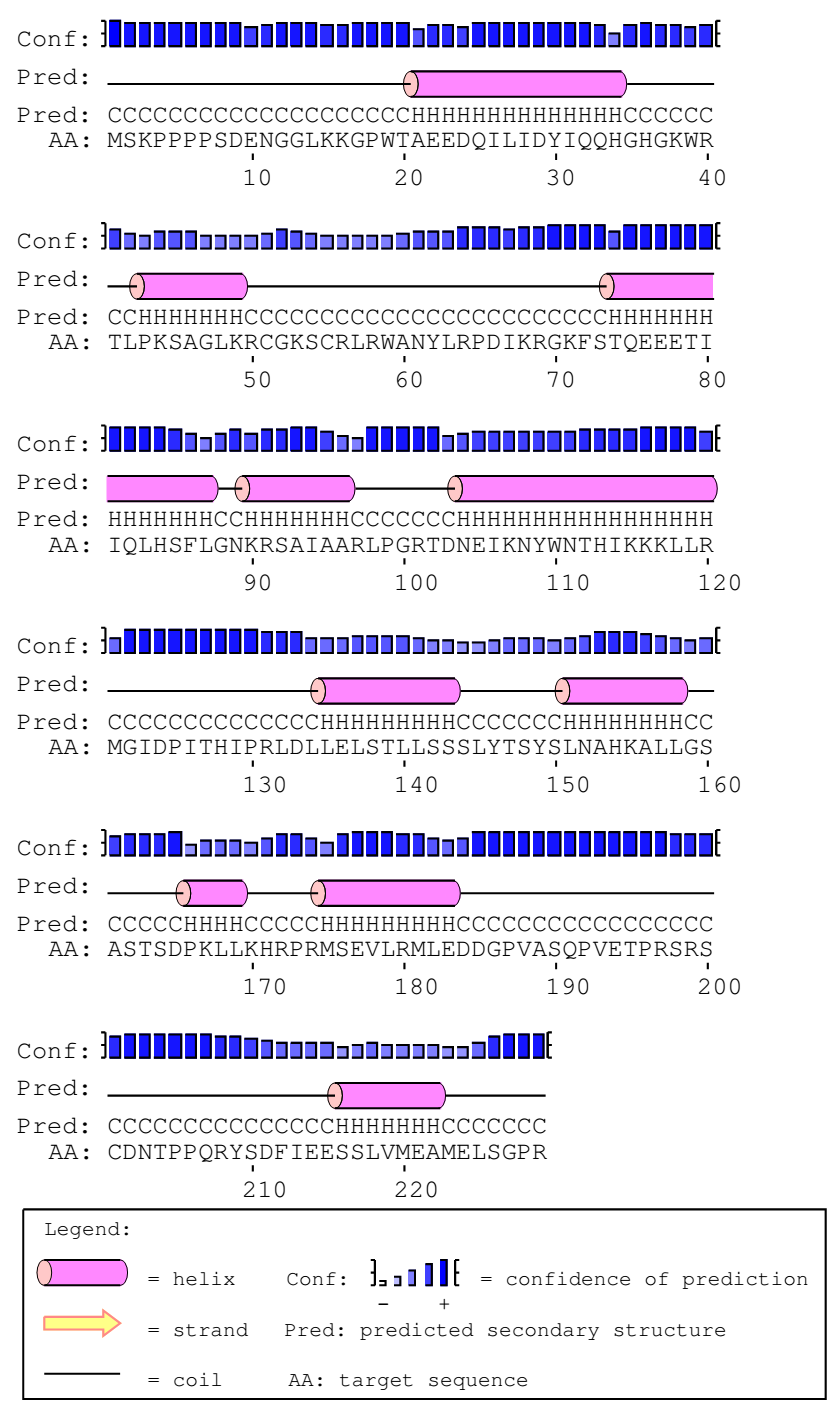
**

S1 B Loop to Helix Ratip C S2: Solvent Accessibility


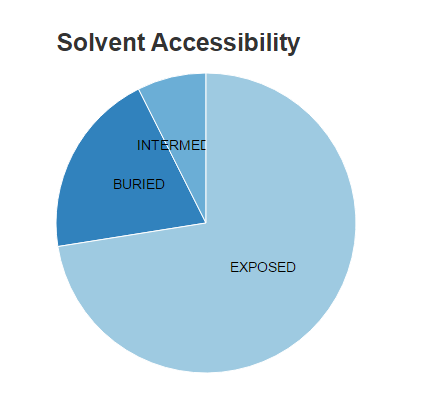

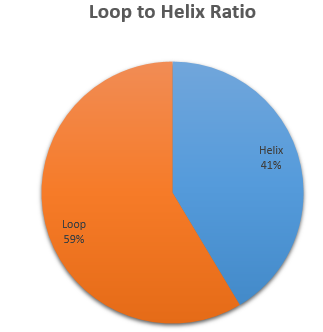


**S3**

**
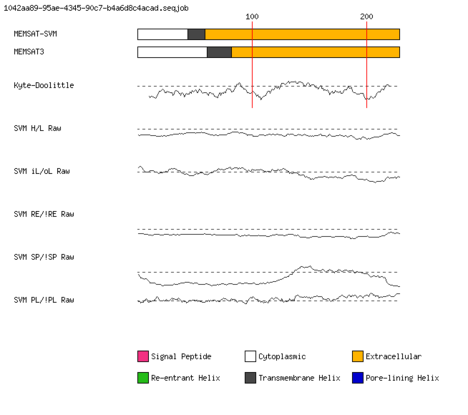
**


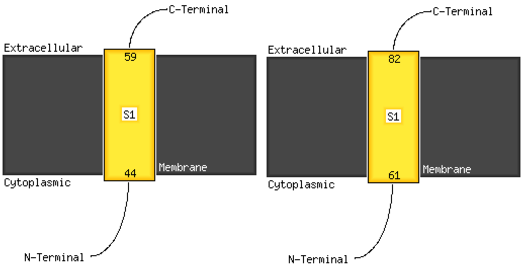


**Supplementary file 1 (B)** 3D-structural models of *RMYB* gene by using different software

3D Jigsaw


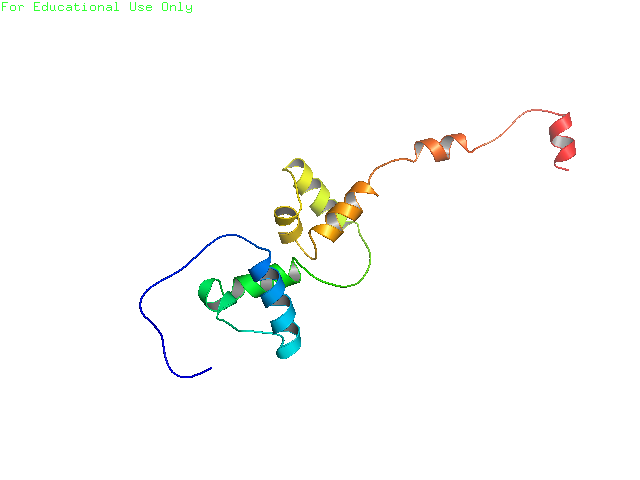


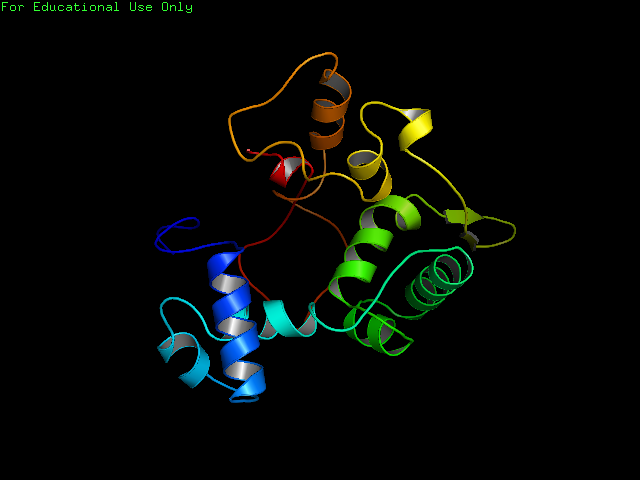
I-Tasser (Model 1)

I-Tasser II


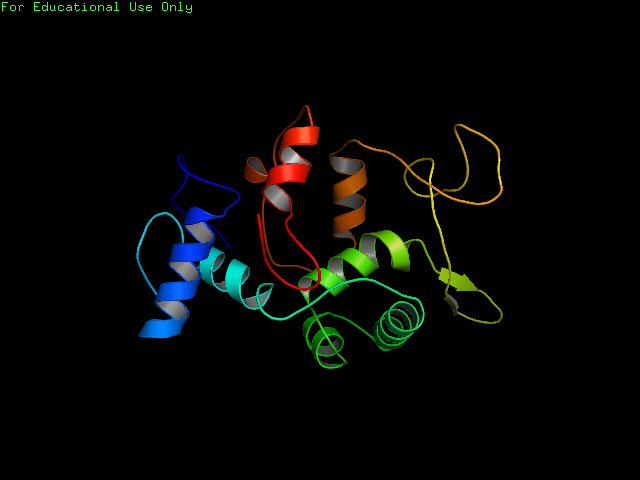


I-Tasser III


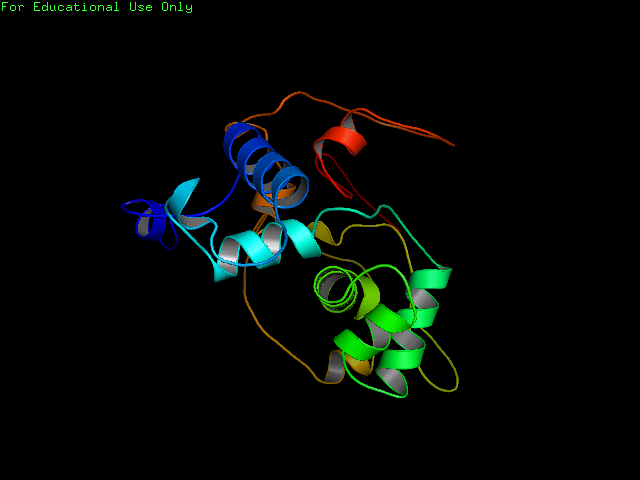


I-Tasser IV


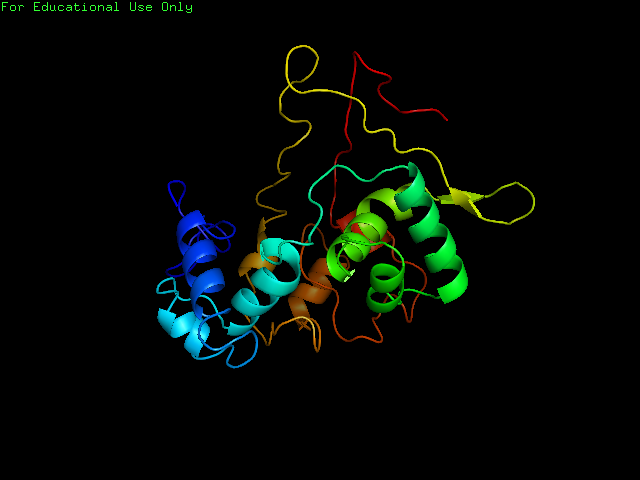


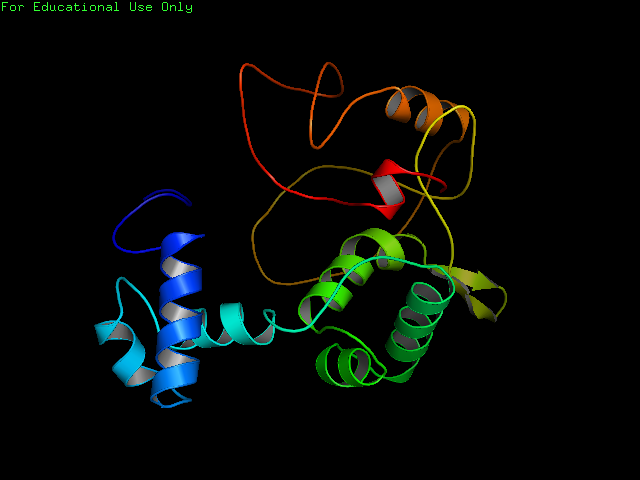
I-Tasser V


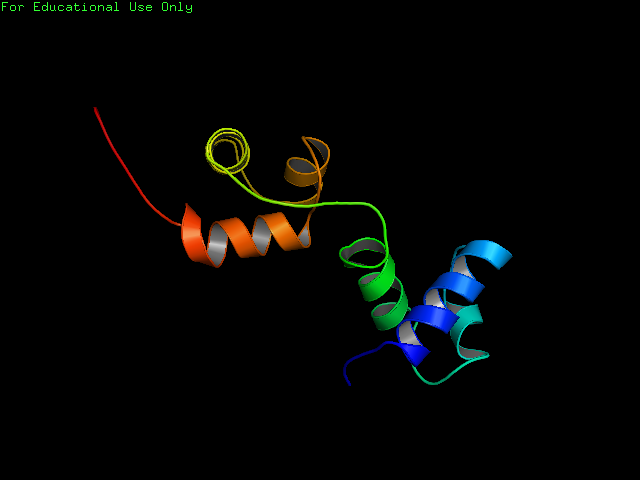
Swiss Model 1

Raptor X


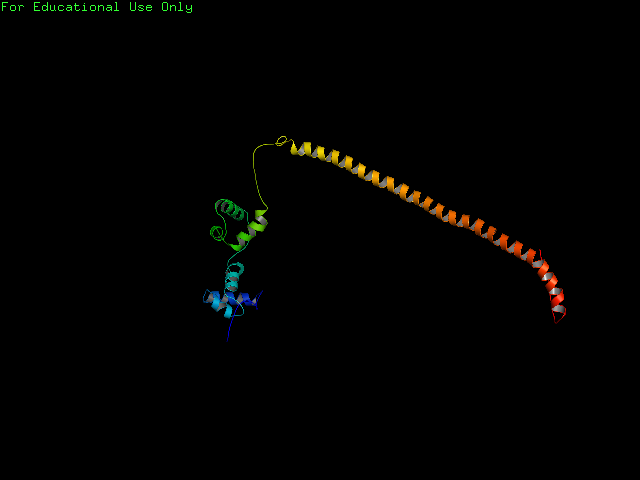


PHYRE 2


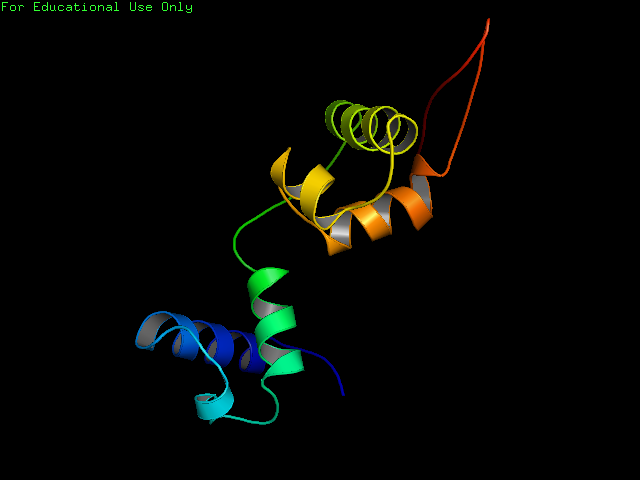


**Supplementary file 1 (C)** Verification of 3D model of *RMYB* gene

|  | Number of residues | | |  |
| --- | --- | --- | --- | --- |
| SERVER NAME | favored region | allowed region | outlier region | Verify 3D result |
| **jigsaw 3D** | | | | |
| Model-1 | 82.9% | 10.7% | 9% | 35% |
| **I-TASSER SERVER** | | | | |
| Model-1 | 73.6% | 21.6% | 4.8% | 35% |
| Model-2 | 74.9% | 21.1% | 4.0 | 50% |
| Model-3 | 74.0% | 16.3% | 9.7% | 21% |
| Model-4 | 72.2% | 20.7% | 7.0% | 23% |
| Model-5 | 74.0% | 18.9% | 7.0% | 33% |
| **SWISS-MODEL SERVER** | | | | |
| Model-1 | 82.2% | 15.0% | 2.8% | 31% |
| Model-2 | 89.7% | 7.5% | 2.8% | 24% |
| Model-3 | 84.1%) | 14.0% | 1.9% | 24% |
| **Phyre-2 SERVER** | | | | |
| Model-1 | 80.2% | 15.5% | 4.3% | 28% |
| Raptor X Protein Modeling Server | | | | |
| Model-1 | 93.0% | 4.4% | 2.6% | 57% |

**Supplementary file 1 (D) :**

**3.33 Transformation of *Gossypium hirsutum* L. CV CIM 496**

3.33.1 Delinting of Cotton Seeds

To remove lint from cotton seeds (*Gossypium hirsutum* L CV CIM 496), concentrated H_2_SO_4_ (95-98%) was used at the rate of 100 mL/kg of seeds. The seeds were continuously stirred with the help of spatula after adding H_2_SO_4_ for 10-15 minutes until all lint was removed from seeds and shiny surface of seeds appeared. Seeds were washed 5-6 times with tap water to remove the acid completely. The seeds, which floated at the surface of water, were removed.

3.33.2 Seed Sterilization

The delinted washed seeds were sterilized by adding few drops of Tween 20 in water washed by vigorous shaking. For surface sterilization, seeds were dipped in 0.1% HgCl_2_ and 0.1% SDS for 15 minutes with constant shaking. After that seeds were washed five times with autoclaved distilled water. The whole washing was done in the laminar air flow cabinet to maintain the sterilize conditions. Then the seeds were soaked in autoclaved distilled water for one hour. After one hour excess water was removed and the seeds were kept in the dark for germination at 30°C overnight.

## 3.34 Transformation of Cotton var CIM -496 with *RMYB* Gene

After the vector transformation and confirmation, mature embryos of local cotton variety CIM 496 were transformed with plasmid DNA *RCU* containing salt resistant gene (*RMYB*). The method used for this purpose was shoot cut apex method (CEMB modified shoot apex cut method as done by Jean *et al.*, (1991). The overall schematic procedure has been described in (Fig. 3.11).

**3.34.1 Isolation of Embryo**

Next day, with a forceps testa of the seeds were removed carefully and cotyledonary leaves were excised with surgical blades. Mature cotton embryos were isolated from the germinating seeds. The isolated cotton embryos were kept on moist filter paper so that they may not become dry.

**3.34.2 Medium Preparation**

To culture the transformed embryos, MS Murashige and Skoog, (1962) media broth (Appendix-9) was used. The medium was sterilized at 121ºC and 15 lbs psi for 20 minutes in an autoclave chamber. Autoclaved medium was allowed to cool down to 50°C and antibiotic cefotaxime were added as 50µg/mL and 250µg/mL respectively to the medium for selection of transformants. Media was poured in glass culture jars and petri plates to solidify at room temperature.

**3.34.3 Bacterial Inoculum Preparation**

*Agrobacterium* strain *GV1301* containing plasmids *RCU* from glycerol stock stored at –70°C was streaked on solidified agar medium containing kanamycin 50µg/mL and incubated for 24-48 hours at 28^o^C. Single colony was picked and inoculated 10mL of YEP (Appendix-9) broth containing 50µg/mL of kanamycin in 50 mL culture tube. The samples were incubated on rotary shaker at temperature 28ºC for 24 hours with 200rpm. After incubation period bacterial culture was centrifuged at 3000xg for 15 minutes. Supernatant was discarded and pellet was resuspended in 10mL of MS broth.

**3.34.4 *Agrobacterium* Culture Treatment**

Shoot tips of the isolated embryos were injured by using sterile blade held on petri plate shoots were highlighted by using light microscope. After cutting, embryos were immediately shifted to the *Agrobacterium* inoculum suspension for treatment with bacterial culture and incubated for 1 hour on a rotary shaker at very slow speed. Total about 3, 000 embryos were used in the transformation experiments.

**3.34.5 Co-cultivation**

After bacterial inoculum treatment, the culture was removed and embryos were shifted to MS + kinetin1mg/mL medium and cocultivated for 72 hours. At this stage antibiotics were not added to the media. The cultures were kept in growth room at a temperature of 25°C± 2°C and a photoperiod of 16 hours light and 8 hours dark.

**3.34.6 Selection of Transformants**

The survival percentage of the embryos at different growth stages i.e. After co-cultivation, shoot and root formation were observed on MS media (Appendix-9).

**3.34.7 Transient Expression of *GUS* Gene**

Transient expression of *Gus* gene was studied through histochemical GUS assay. GUS solution was prepared containing 25mg/L X-gluc, 10mM EDTA, 100mM NaH_2_PO_4_, 0.1% Triton X-100 and 50%methanol, (pH was adjusted to 8.0). The GUS solution was protected to light exposure. After 72 hours of co-cultivated embryos were dipped in GUS solution in an eppendorf and kept at 37ºC overnight and viewed under microscope for blue spots. The tissues were incubated with X-Glu solution at 37ºC for 16 hours. The blue spots were observed. Transformed plant tissues were checked with GUS assay using non-transformed plants as negative control.

### 3.34.8 Plants subculture

After three days (72hrs) of co-cultivation, plantlets were shifted to medium i.e. MS and ceftriaxone (250μg/mL). Medium was also supplemented with different growth hormones i.e. Kinetin (1mg/mL), Zeatin (1mg/mL) and BAP (1mg/mL).

3.34.9 Transformation Efficiency

Total 3,000 embryos were used in the transformation experiments. The transformation efficiency after eight weeks of transformants growing on selection medium was calculated. This was evaluated on 50 mg/mL Kanamycin.

### 3.34.10 Root Formation

After 6-8 weeks, the selected plants with well-developed shoots were sub cultured to selection free medium. At this stage MS medium as supplemented with different growth hormones in combinations or alone to observe the rooting (kinetin 1mg/mL+1mg/mL), (kinetin1mg/mL+IBA1mg/mL), (IAA1mg/mL). Plants were continued to subculture on the selection free medium supplemented with growth hormones for 4-6 weeks.

3.34.11 Transformation of Transgenic Plants to Soil

Transformed plants were shifted to prepared soil mixture from glass culture tubes to soil. Soil mixture contained clay, sand and peat moss in ratio of 1:1:1, mixed well, moistened properly with dH_2_O, filled in a bag and autoclaved. After soil autoclaved was allowed to cool and filled in plastic pots having at the bottom drain hole.

Then rooted shoots were taken out of the glass tubes, the root was washed with autoclaved water, dipped into IBA (1mg/mL) and then planted into the pot filled with soil mixture. None transformed were shifted to soil without IBA treatment and pots were covered with polythene bags. Transgenic Plants were kept in a growth room at 30ºC+2ºC for 16 hours photoperiod with light intensity (250-300µmol m^-2^ S^-1^). After 3-4 days polyethylene covers were removed occasionally two times daily. After 1-2 weeks the cover were removed completely. The transgenic plants were ready to shift to glass house.

**Procedure Optimized Parameters**

Seeds Delinting

Surface sterilization

Incubation for germination

(Overnight)

Isolation of embryos

Shoot apex cutting and Co-cultivation

(72hours)

Callus induction

Media S-4

Shootinitiation

2 weeks

Shoot outgrowth 2 weeks

Shoot elongation

Growing in selection media

(4 – 6 weeks)

Shifting to soil then glasshouse

Analysis of transgenic plants

Supplementary Table 1: List of Arbitrary and Anchored primer used for differentially expressed transcript identification

| **Arbitrary sequence primers** | | **Anchored sequence primers** | |
| --- | --- | --- | --- |
| P1 | 5'ATTAACCCTCACTAAATGCTGGGGA-3' | T1 | 5'-CATTATGCTGAGT-GATATCTTTTTTTTTAA-3 |
| P2 | 5'-ATTAACCCTCACTAAATCGGTCATAG-3' | T2 | 5'-CATTATGCTGAGTGATATCTTTTTTTTTAC-3' |
| P3 | 5'-ATTAACCCTCACTAAATGCTGGTGG-3' | T3 | 5'-CATTATGCTGAGTGATATCTTTTTTTTTAG-3' |
| P4 | 5'-ATTAACCCTCACTAAATGCTGGTAG-3' | T4 | 5'-CATTATGCTGAGTGATATCTTTTTTTTTCA-3' |
| P5 | 5'-ATTAACCCTCACTAAAGATCTGACTG-3' | T5 | 5'-CATTATGCTGAGTGATATCTTTTTTTTTCC-3' |
| P6 | 5'-ATTAACCCTCACTAAATGCTGGGTG-3' | T6 | 5'-CATTATGCTGAGTGATATCTTTTTTTTTCG-3' |
| P7 | 5'-ATTAACCCTCACTAAATGCTGTATG-3' | T7 | 5'-CATTATGCTGAGTGATATCTTTTTTTTTGA-3' |
| P8 | 5'-ATTAACCCTCACTAAATGGAGCTGG-3' | T8 | 5'-CATTATGCTGAGTGATATCTTTTTTTTTGC-3' |
|  |  | T9 | 5'-CATTATGCTGAGTGATATCTTTTTTTTTGG-3' |
|  |  | B1 | 5'-AAGCTTTTTTTTTTTTTA-3' |
|  |  | B2 | 5'-AAGCTTTTTTTTTTTTTG-3' |
